# An insight into osteoarthritis susceptibility: Integration of immunological and genetic background

**DOI:** 10.17305/bjbms.2020.4735

**Published:** 2021-04

**Authors:** Debora Stefik, Vladimir Vranic, Nemanja Ivkovic, Dzihan Abazovic, Dusan Maric, Danilo Vojvodic, Gordana Supic

**Affiliations:** 1 Institute for Medical Research, Military Medical Academy, Belgrade, Serbia; 2 Clinic for Orthopedic Surgery and Traumatology, Military Medical Academy, Belgrade, Serbia; 3 Renova Clinic, Belgrade, Serbia; 4 Institute for Child and Youth Health Care of Vojvodina, Novi Sad, Serbia; 5 Medical Faculty of Military Medical Academy, University of Defense, Belgrade, Serbia

**Keywords:** Osteoarthritis, immunology, genetic polymorphisms, genetic susceptibility

## Abstract

Osteoarthritis (OA) is a progressive degenerative disease that affects all synovial joints, causing the disability of the main locomotor diarthrodial joints. OA pathogenesis is caused by a complex interplay between a number of genetic and environmental risk factors, involved in the early onset and progression of this chronic inflammatory joint disease. Uncovering the underlying immunological and genetic mechanisms will enable an insight into OA pathophysiology and lead to novel and integrative approaches in the treatment of OA patients, together with a reduction of the disease risk, or a delay of its onset in susceptible patients.

## INTRODUCTION

**FIGURE 1:**
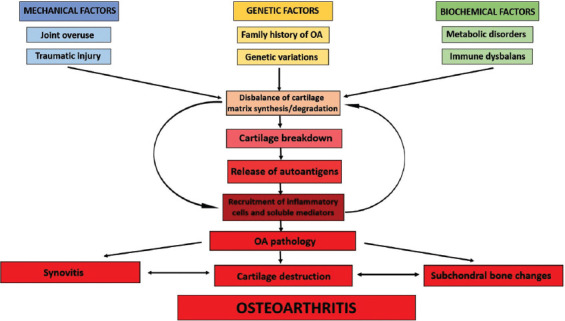
Factors implied in osteoarthritis onset, central events, and disease features. OA: Osteoarthritis.

Osteoarthritis (OA) is the most common chronic ­inflammatory joint disease that leads to chronic conditions and disabilities as a result of various pathological changes, including synovial inflammation, cartilage degradation, subchondral bone alterations, and impairment of the supporting musculature. It is reported that 10% of men and 18% of women over 60 years are affected by this joint disabling disorder worldwide [1]. The prevalence and incidence of OA increase with age and are higher among women, and the prevalence is significantly rising among postmenopausal women [2]. This degenerative disease predominantly involves weight-bearing joints of lower extremities (the hip, knee, and ankle), exposed to constant and excessive mechanical loading. Approximately 80% of OA patients have a reduced range of joints’ motion, while 25% of patients have impaired quality of life due to limitations in activity, pain, deformity, and swelling [3]. Moreover, this condition has a considerable personal and social impact due to the aging of the global population and the increase in obesity rates [4]. The complex interactions between a number of genetic and environmental risk factors, including developmental disorders, obesity, metabolic factors and prior joint injuries, contribute to OA initiation and progression (Figure 1) [4].

There is a continuous effort for discovering the predisposing loci in OA genome-wide association studies (GWASs) and their functional relevancy, as well as their interaction with the known risk-conferring etiological factors. Although there is limited information available from family studies, genetic factors are well recognized as contributing factors to OA susceptibility [5]. Genomic studies have shown that genetic polymorphisms, predominantly single nucleotide polymorphisms (SNPs), are linked with OA occurrence, but could also be associated with OA progression or could delay/enhance its onset in susceptible individuals. Previous studies reported SNPs in various genes as genetic risk markers for OA, including genes encoding cartilage and bone metabolism mediators, such as vitamin D receptor (VDR) [6], pro-inflammatory cytokines, different members of the metalloproteinase family [7,8], and related genes, such as growth differentiation factor 5 (GDF5) [9]. However, the genetic background of OA has complex origins and effects, and many studies show conflicting results.

## IMMUNOLOGY OF OA

A variety of immune mediators and metabolic intermediates affect the homeostasis of joint structures, causing the imbalance between anabolic and catabolic processes, which leads to the OA development and progression [10].

One of the risk factors for OA degenerative processes is an inflammatory reaction. Elevated levels of inflammatory mediators are detected in almost every OA joint tissue – ­synovial fluid, synovial membrane, subchondral bone, and cartilage [11]. A critical factor in OA development is the damage of cellular and extracellular matrix (ECM) upon joint injuries, chronic mechanical insults, trauma, or aging. The endogenous damage-associated molecules, released upon cellular stress or tissue injury, lead to the activation of innate immunity through receptor-dependent mechanisms, pattern-recognition receptors, and consequent inflammatory reaction [12]. When triggered, the inflammatory response leads to the upregulation of mediators that further propagate inflammation and cause disbalance of homeostasis and pro-catabolic reprogramming [13].

## ROLE OF CYTOKINES IN OA

The initiation and maintenance of the inflammatory processes in OA joints are controlled by different pro- and anti-inflammatory cytokines. Interleukin (IL)-1β and tumor necrosis factor-α (TNF-α), key pro-inflammatory cytokines that exhibit a synergistic effect, are elevated in synovial fluid, synovial membrane, cartilage, and the subchondral bone of OA joints [14].

IL-1β-mediated catabolism is considered as the key pathophysiological process in the course of OA [15]. An important mechanism in the OA pathogenesis is IL-1β-induced upregulation of matrix metalloproteinases (MMPs), the group of enzymes involved in the degradation of collagen and other proteins in the ECM. Interstitial collagenase (MMP-1), stromelysin-1 (MMP-3), and collagenase 3 (MMP-13) appear to degrade the components of the cartilage ECM in arthritis [16]. The A disintegrin and metalloproteinase with thrombospondin motifs (ADAMTS) metalloproteinase family, aggrecan degradation molecules, are also involved in IL-1β-induced cartilage matrix degradation [14]. Production of ADAMTS proteases upon IL-1β stimulation was demonstrated both in human OA chondrocytes [17] and synovial fibroblasts [18]. IL-1β is also associated with the regulation of apoptosis in cultured human OA chondrocytes, through the upregulation of Bcl2 family [19] and the activation and/or production of the oxidative stress intermediates nitric oxide (NO), reactive oxygen species (ROS), and prostaglandin [20]. Also, IL-1β suppresses the anabolic mechanisms in cartilage tissue by repressing the gene expression of ECM constituents (aggrecan and type II collagen) [21].

TNF-α is another important pleiotropic cytokine involved in OA pathogenesis. It plays a dual role in the regulation of immune responses, acting both as a pro-inflammatory mediator, through the initiation of an inflammatory response, and as an immunosuppressive mediator, regulating the duration of the inflammatory response that limits the tissue damage [13]. Similarly to IL-1β, TNF-α induces the expression of matrix-remodeling enzymes in chondrocytes [13,22], and its overproduction is also associated with increased oxidative stress [20].

The levels of key cytokine receptors (IL-1RI, IL-1RII, and TNF receptor p75) are reported to be significantly higher in both OA cartilage and synovium [23], indicating the higher sensitivity of joint cells to stimulation by those cytokines.

IL-6, one of the highly elevated pro-inflammatory cytokines in OA, has a complex role in OA as a modulator of inflammatory reaction. Binding to a membrane-bound IL-6 receptor (mIL-6Ra) is involved in one of the biological functions of IL-6, which is known as the “classical” pathway. This results in downstream activation of the Janus kinase/signal transduction and activators of transcription (JAK/STAT) signaling pathway, which promotes STAT proteins to act as transcription factors in an inflammatory reaction. Another pathway, based on the interaction of IL-6 with a soluble IL-6 receptor (sIL-6R) and referred to as the “trans-signaling”, is involved in the modulation of the MMP-9 activity, crucial for the cartilage degeneration in OA [24]. A number of studies in OA have shown that IL-6 production is upregulated by IL-1β and MMPs [25]. An increased concentration of circulating IL-6, as well as a high body mass index, was associated with the development of knee OA [26]. Furthermore, higher levels of IL-6 were detected in the synovial fluid of obese OA patients, compared to normal-weight patients [27]. The elevated levels of IL-6 are manifested by abundant infiltration of T-cells in the synovial tissues of OA patients, enhanced production of matrix-degrading enzymes, and inhibition of the ECM constituent synthesis [28].

Several studies also showed a positive association between arthritic disorders and the diverse roles of IL-17 [29]. Patients with OA, especially those with advanced OA stages, are reported to have significantly higher levels of IL-17 in the serum and synovial fluid, compared to healthy controls [30,31]. Besides, IL-17 contributes to cartilage degeneration and synovial infiltration in OA by stimulating chondrocytes and synovial fibroblasts to release IL-8, growth regulated oncogene-α (GRO-α) and monocyte chemoattractant protein-1 (MCP-1) and, to a lesser extent, by inducing the IL-1β synthesis by chondrocytes [32]. Moreover, IL-6 and IL-17 act synergistically with IL-1β and TNF-α in inducing the release of inflammatory mediators in affected joint tissues in OA [33].

Several other cytokines and chemokines, such as IL-8, IL-15, and IL-18, have also been identified as elevated in the constituent cells of the joint [14]. These mediators have a role in OA pathogenesis by favoring the persistent inflammation in joints.

It is known that IL-1β plays a substantial role in the course of OA through transcriptional activation of inflammation-related genes [34]. The polymorphisms in the gene encoding IL1-β, rs16944 (-511, G>A) and rs1143634 (+3594, C>T), were previously associated with an increased risk for OA (Table 1). A study on the Croatian Caucasian population reported an association between rs16944 and rs1143634 in female OA patients, indicating these polymorphisms as possible gender-dependent factors for OA susceptibility [35]. Recently, the positive association of the IL-1 gene cluster (IL-1α [*IL1A*], IL-1β [*IL1B*], and interleukin-1 receptor antagonist [IL-1RA, *IL1RN*] genes) with severe knee OA was reported [36].

**TABLE 1:**
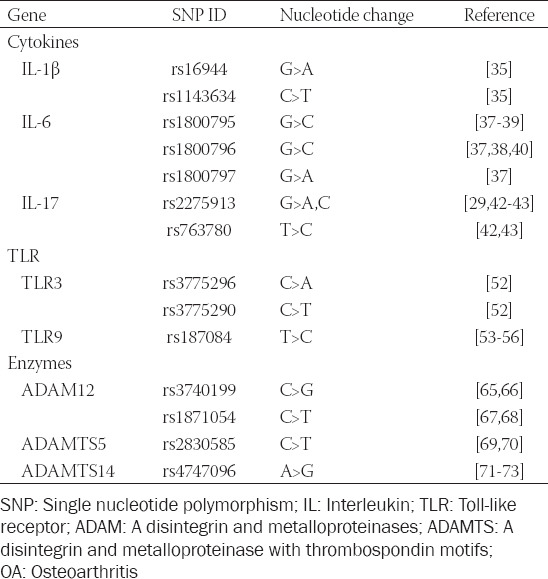
Single nucleotide polymorphisms in immune-related genes linked with OA susceptibility/severity

Polymorphisms in the gene coding for the pro-inflammatory cytokine IL-6 are established as one of the key genetic factors for OA susceptibility. The IL-6 promoter variants rs1800797, rs1800796, and rs1800795 (-597, G>A; -572, G>C; and -174, G>C, respectively) were reported to affect both the transcription and secretion of IL-6 in OA [37]. The IL-6 SNP rs1800795 was linked to hip and knee OA susceptibility and severity [38,39], while another IL-6 polymorphism rs1800796 was indicated as a protective factor for the occurrence and severity of hip and knee OA in the elderly [38,40]. In addition, transcriptional regulation of the IL-6 rs1800795 is controlled by two other polymorphisms, rs1800796 and rs1800797, indicating the complex and cooperative regulation of various genetic polymorphisms in the modulation of IL-6 expression [41].

Several studies investigated the role of IL-17 in OA pathology, but data on this cytokine gene polymorphisms and OA susceptibility are still inconsistent. The study by Bai et al. reported the IL-17 polymorphisms rs2275913 (-197, G>A,C) and rs763780 (7488, T>C) as closely linked to knee OA susceptibility [42]. On the other hand, the study by Jiang et al. reported a significant association of knee OA with rs2275913 but not with rs763780 [43]. In addition, conflicting data regarding rs2275913 and OA susceptibility are available, since there is no evidence of an association between this genetic variation and hip/knee OA in a study by Vrgoc et al. [29].

## INVOLVEMENT OF TOLL-LIKE RECEPTORS (TLRs) IN OA

TLRs represent a group of highly conserved innate immunity receptors, involved in the initiation of protective responses to pathogens. TLRs have a pivotal role in the detection of viral, bacterial, and fungal infection-associated molecular patterns (pathogen-associated molecular patterns, PAMPs). TLRs are engaged in the activation of diverse signaling pathways and transcriptional activation of various adapter molecules, kinases, and transcription factors such as nuclear factor-kB (NF-κB) and interferon (IFN) regulatory factors (IRFs). The downstream TLR signaling, activated by ­ligand-receptor interactions, results in initiation and perpetuation of an inflammatory immune response, through transcriptional activation of the genes encoding cytokines (IL-1, IL-6, IL-8, IL-12, and TNF-α), chemokines (C-C motif chemokine ligand 5, CCL5), and cellular adhesion molecules (intercellular adhesion molecule-1 [ICAM-1] and its counterpart lymphocyte function-associated antigen-1 [LFA-1]) [44]. Damage-associated molecular patterns (DAMPs) are endogenous TLR ligands that are also generated during inflammation and are associated with joint damage processes such as matrix degradation and bone remodeling [45]. A wide range of DAMPs within the OA joint initiates TLR-dependent response and leads to coordinated recruitment and intercellular communication among immune cells, such as macrophages and neutrophils, and resident cells that create an inflammatory environment [46]. The synthesis of pro-inflammatory cytokines through activation of different TLRs (TLR1, TLR2, TLR3, TLR4, and TLR9) is detected in arthritic synovial fibroblasts and articular chondrocytes [47-49]. Endogenous activators of TLR-mediated immunity such as components of the cartilage ECM (low-molecular-weight hyaluronan, heparan sulfate, biglycan, fibronectin, and tenascin c) and alarmins, such as S100 proteins and high-mobility group protein B1 (HMGB1), were found in OA synovial fluid [50]. Sillat et al. also reported a link between increased expression of TLR1, TLR2, TLR4, and TLR9 with the severity of OA [47].

One feature of OA is excessive activation of TLR and their downstream signaling molecules that contribute to OA pathogenesis through systemic sustaining of low-grade inflammation. Inflammation-related mediators (NO, prostaglandins) and inflammatory cytokines (IL-1β, TNF-α, IL-6, and IL-8), which induce catabolic responses within cartilage, are actively produced by OA chondrocytes [25]. The study on catabolic pathways mediated by TLRs in OA cartilage showed abundant expression of TLR-2 and TLR-4 in OA cartilage lesions [51]. Sillat et al. demonstrated an aberrant TLR profile in osteoarthritic cartilage with upregulated expression of not only TLR2 and TLR4 but also TLR1 and TLR9 in cartilage lesions of advanced OA [47].

The genetic variability of TLRs could lead to the activation of the downstream signaling and subsequent elevation of cytokine levels, such as IL-1β, IL-6 and IL-17, whose roles in OA pathophysiology are thoroughly examined (Table 1). However, there are limited data regarding the genetic polymorphisms of the TLR receptor family in OA. Association between the TLR-3 promoter polymorphisms rs3775296 (-7, C>A) and rs3775290 (1377, C>T) and knee OA susceptibility was found [52]. Several studies reported rs187084 (-1486, T>C) polymorphism of the *TLR9* gene as a predisposing genetic marker for knee OA development in Chinese [53,54] and Turkish population [55]. The polymorphism rs187084 was also found to be significantly associated with hip OA risk [56].

## REMODELING OF THE ECM IN OA

**FIGURE 2:**
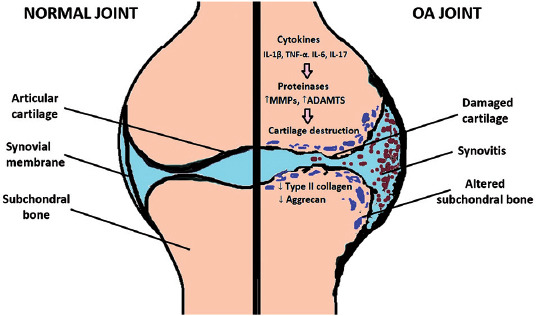
Normal joint and joint in osteoarthritis. OA: Osteoarthritis; MMPs: Matrix metalloproteinases; ADAMTS: A disintegrin and metalloproteinase with thrombospondin motifs; IL: Interleukin; TNF-α: Tumor necrosis factor α.

The dysregulated expression of matrix-degrading enzymes that leads to degradation of the cartilage is considered as a hallmark of OA. The degradation of collagen and aggrecan molecules, induced upon the expression of inflammatory mediators such as IL-1β and TNF-α, is directly associated with the progression of OA [57] (Figure 2). MMPs and the related families that share the metalloproteinase domain, membrane-anchored A disintegrin and metalloproteinases (ADAMs) and the secreted ADAMs with thrombospondin repeats (ADAMTSs), play an essential role in the ECM and the cartilage degeneration during OA progression. Members of ADAMS superfamily have diverse functions in cellular processes related to immune response, such as cellular adhesion, cell signaling, and the cleavage of the extracellular domains of membrane-associated proteins, a process known as “ectodomain shedding” [57,58].

ADAM10 and ADAM12 are identified as osteoclastic cell differentiation factors [58]. ADAM10 is considered as a progenitor cell fate determination factor during bone development and remodeling [59]. ADAM15 is upregulated in chondrocytes from the early stages of OA and is involved in cartilage remodeling by exerting anti-apoptotic effects [60]. The association between the radiographic features of knee OA and ADAM12 at both gene and protein levels [61] indicated the potential role of ADAM12 in the development of OA.

The ADAMTS group of zinc-dependent proteases is implicated in physiological and pathological tissue remodeling and inflammation [62]. ADAMTS4 and ADAMTS5, also known as aggrecanases 1 and 2, respectively, have been reported as crucial for cartilage aggrecan degradation in OA. ADAMTS4 and ADAMTS5 have differential regulation. While the *ADAMTS4* gene expression is found to be upregulated as the result of pro-inflammatory IL-1β, TNF-α, and TGF-β activity, *ADAMTS5* does not respond to this pro-inflammatory cytokine stimulation [63]. A study on the expression profiling of ECM degradation mediators in OA demonstrated a dysregulated pattern of ADAMTS genes expression, with upregulated ADAMTS2, -12, -14, and -16 in OA cartilage, compared to normal cartilage samples [64].

Polymorphisms in genes encoding metalloproteinases, ECM-degrading proteases, have also been identified as genetic risk factors in OA (Table 1). Several polymorphisms within the *ADAM12* gene have been reported to have a significant impact on OA occurrence. The ADAM12 rs3740199 polymorphism has been associated with knee OA in male patients [65,66], indicating that inflammatory responses in OA differ between genders. The association of the other *ADAM12* polymorphism, rs1871054, has also been linked to knee OA susceptibility and severity in the Asian population [67], but not in the Caucasian population [68].

Genes that encode ADAMTS family members are also considered as novel OA candidate genes, but with inconsistent findings in different populations. A positive association between the *ADAMTS5* rs2830585 genetic polymorphism and OA development was found in the Chinese population [69], while in the Turkish population no association of rs2830585 with OA susceptibility was observed [70]. The involvement of *ADAMTS14* genetic polymorphisms in OA development has been also noted recently [71]. A positive association between the *ADAMTS14* gene polymorphism rs4747096 and an increased risk of OA was reported [72], with a possible gender-dependent association, especially for knee and hand OA susceptibility [71,73].

## FUTURE PERSPECTIVES

Multifactorial diseases, such as OA, are challenging to study and treat due to the involvement of polymorphisms in multiple genes and association with various environmental causes. Elucidation of the diverse immunoregulatory network along with the genetic architecture of OA would provide an insight into mechanisms underlying this complex disease as well as gene-environment interactions.

There is a continuous effort for discovering novel predisposing loci in OA GWAS studies and functional relevancy of genes previously implicated in OA. However, the genetic background of OA is complex, and inheritance patterns that could be responsible for triggering disease onset and/or progression, are generally unknown. Moreover, gene-environment interactions most likely play an important role in the development of OA and significant clinical variations, including joint pain, stiffness, and locomotor restriction.

Establishing the genetic network of OA in the future will provide guidance for precision medicine and identify the ­predisposing genetic risk factors. Also, revealing possible therapeutic targets in the treatment of OA and further development of immunotherapies will provide an optimal strategy for personalized approaches in OA therapy, based on the patient’s genotype [74]. Further genetic studies identifying the underlying mechanisms of OA could also give recommendations for individualized nutrition that may slow down the progression, improve symptoms, and prevent or delay surgical treatment in patients with advanced disease. Poor diet and adverse lifestyle factors such as low physical activity, obesity, high alcohol intake, and smoking are major risk factors for developing certain complex diseases, including OA. What is more, emerging evidence indicates that various bioactive dietary components, as well as caloric intake, could play a protective role in the immunomodulation through their anti-inflammatory and antioxidative effects [75]. Classification of relevant genetic changes may enable early identification of potential therapeutic targets in susceptible individuals and personalized approaches to achieve optimal clinical care. There are growing efforts to develop relevant scores to achieve an optimal diagnosis and clinical care of patients with OA [76]. Several protein-based markers from serum and urine have been assessed as indicators of OA burdens [77,78]. Moreover, a study of knee OA [26] assessed a serum IL-6 assay and clinical features such as patient’s age and BMI in the stratification of the patient’s risk.

Although there are a number of recent studies on genetic polymorphisms in OA [79], the actual SNPs and genes are still not included in clinical protocols of OA patients’ treatment. Integration of biochemical and genetic biomarkers, as well as clinical data, will improve clinical care of OA patients, beyond reporting associations of biomarkers with OA.
